# Supplementary material for: Patterns of Intron Gain and Loss in Fungi
Source: PLoS Biol. 2004 Nov 30;2(12):e422. doi: 10.1371/journal.pbio.0020422 (PMC532390; doi:10.1371/journal.pbio.0020422)
Supplement: Table S1 — Also available at http://genes.mit.edu/NielsenEtAl/. (4.3 MB ZIP). [file pbio.0020422.st001.zip › NielsenEtAl/html/45.html]

AN5019.1.NCU08434.1.MG05888.1.FG07880.1


```
 CLUSTAL W (1.82) Multiple Sequence Alignments - Introns Inserted


Sequence 1: NCU08434.1	401 aa
Sequence 2: FG07880.1	404 aa
Sequence 3: MG05888.1	399 aa
Sequence 4: AN5019.1	438 aa
Alignment Length: 445 aa
Number Identitical Residues: 168 aa
Alignment Score (without introns) 9762


MG05888.1 	------------------------------~------------MAPPFRAEHVGSLLRPQ
NCU08434.1	------------------------------~-------------MAPFRVEHMGSLLRPQ
FG07880.1 	------------------------------~----------MAPLQPFRAEHMGSLLRPQ
AN5019.1  	MARLILAPHSFSLVSFRNTLLSLLTSTFTW2IIVSQNFTMSLHRCPPFRVEHLGSLLRTK
          	 :    :. : :  :  .:  :  ::: :     :.. : :     ***.**:*****.:

MG05888.1 	DLLDVRASIAAGG---DDSKLAGVEKAAVKEVVDLQRSLGFKPVTSGEYVRTR~FWGLMW
NCU08434.1	NLLDAREAIRDKGLSPEEAGLPAIEKEAVGNVVKLQQELGFKAVNSGEFNRTR~FWGLMW
FG07880.1 	ALLDVREQIREKGLSPENAGLEAVENEAVKEVVKMQQELGFKAVTSGEFTRTR~FWGLMW
AN5019.1  	ELLDVKTAYENGK--ATKEQLEAVEKKDIKDVVELQKKLRYSALSDGEYCRHM1FWGSFF
          	 ***.:         . .  * .:*:  : :**.:*:.* :..:..**: *   *** ::

MG05888.1 	DEFEGTKRLQDAEASMFRLYHPDVVSLIEQDRKVMPGDSVIAGAKLSHSKDKCKSNLHEL
NCU08434.1	DEFEGSVALQEAEASMFRLYHPDVVSLIEKDRKVMPGDSVIAGAKLRHNREKSKSNLHEL
FG07880.1 	DEFEGSTQLQDAEASLFRLYHPDVVSLIEKDRQVMPGDSVIASSKLSHNPEKSVSNLHEL
AN5019.1  	PGLEGFDEVSDPSPEVFRPYAPDVAAFLEAGHK--PGESVFCTGKIKHVG---STYVDQF
          	  :**   :.:....:** * ***.:::* .::  **:**:. .*: *      : :.::

MG05888.1 	KLVQENTPKEEWGNIKLTM0ITPAWFHMRYKQGRAYTPEAYANDAEYFADVAKVYQDELQ
NCU08434.1	KLVQQFTPKEEWANIKLTM~ITPAWFHMRYKQGKAYAEGVYANDEEYFADVAKVYQEELK
FG07880.1 	KLVQQFVPKEQWGSIKLTM~ITPAWFHMRYKQGKAYAPEAYKNDEEYFADVAKVYQAELD
AN5019.1  	KFLASLVAPEEVKNLKLTL~AAPNWYHLRYREGYAYPKEVYANVDEYFADIAKAYQDELK
          	*:: . .. *:  .:***:  :* *:*:**::* **.  .* *  *****:**.** **.

MG05888.1 	QLYDAGLRNVQFDDPGLAY1FCSQKFREGWAEDKDNIGEVDDLLDAYIKLYNDSIAKLPS
NCU08434.1	MLYEAGLRNVQFDDPGLAY1FCSEEFRAGYASDPDNTVSIDTLLDSYIKLYNDSLSLLPA
FG07880.1 	MLYKAGLRNVQFDDPGMAY1FCSQKFRDGWAADSDNIGTVDDLLDAYIKLYNNSLSKIPS
AN5019.1  	ILYDAGCRNVQFDDPNLVY1FCSEKMLQGWKEDPLNTLSADETFEKYIKLYNDCLATRPK
          	 **.** ********.:.* ***:::  *:  *  *    *  :: ******:.::  * 

MG05888.1 	DMHTGVHLCRGNFIGGRHFSEGSYDIIAKKLFQNLHVNTFYLEYDTQRAGGFEPLKYLPK
NCU08434.1	DFHTGVHLCRGNFIGGRHFASGAYDVIAQKLFQELNVNTFYLEYDTARAGGFEPLQYLPK
FG07880.1 	DMHTGIHLCRGNFIGGRHFAEGKYDIIAEKLFRDLNVNTFYLEYDTERSGGFEPLEFLPK
AN5019.1  	DFHVGVHLCRGNFVGSRHFSEGGYDRIATKLFKELNVDTYYLEYDTPRAGGFEPLKEVPR
          	*:*.*:*******:*.***:.* ** ** ***::*:*:*:****** *:******: :*:

MG05888.1 	DKNVVVGVISTKLPGLEDKDEVKRRIHQAADFVAEGSGQTREEALKRISVSPQCGFSTHE
NCU08434.1	NKNVVLGVVSTKLRELESKEEVKRRVEEAASWVAKGTGESREEALKRVMVSPQCGFSTHE
FG07880.1 	NKSVVVGVISTKLPQLEDKEEMKKRVLSAADWVAKGSGESQKEALQRVAVSPQCGFSTHE
AN5019.1  	HKSVIFGVVTSKFPQLEDKEEMKKRVYDAAKFIAEGNGITLEQALDQVGVSPQCGFASHR
          	.*.*:.**:::*:  **.*:*:*:*: .**.::*:*.* : ::**.:: *******::*.

MG05888.1 	SGYPLSIDEEKAKLKLVRDIADEIWGEP-
NCU08434.1	SGYPLSEDDQRAKLRLVREVADEIWGEA-
FG07880.1 	SGYPLNLDEEKKKLALVREIADEIWGEA-
AN5019.1  	EGNAIDREGMIKKLELVRAIADDIWPGQL
          	.* .:. :    ** *** :**:**
```
